# Machine learning optimization of bio-sandcrete brick modelling using response surface methodology

**DOI:** 10.1038/s41598-024-54029-5

**Published:** 2024-02-10

**Authors:** Nakkeeran Ganasen, L. Krishnaraj, Kennedy C. Onyelowe, Liberty U. Stephen

**Affiliations:** 1 Department of Civil Engineering, SRM Institute of Science and Technology, Kattankulathur, Chengalpattu, Tamil Nadu India 603203; 2 Department of Civil Engineering, Michael Okpara University of Agriculture, Umudike, Nigeria; 3 Department of Civil Engineering, University of Peloponnese, 26334 Patras, Greece; 4 Department of Civil, School of Engineering and Applied Sciences, Kampala International University, Kampala, Uganda; 5 Department of Civil Engineering, University of Agriculture and Environmental Sciences, Umuagwo, Imo State Nigeria

**Keywords:** Raw grinded groundnut shell, Compressive strength, Cement brick, Response surface methodology, Civil engineering, Structural materials

## Abstract

In this study, raw grinded groundnut shell (RGGNS) was used as a fine aggregate in the brick industry to reuse agricultural waste in building materials. In this study, an experimental approach was used to examine a new cement brick with raw groundnut shells integrated with compressive strength, water absorption and dry density optimization utilizing response surface methodology (RSM). The raw ground-nut shell content improved the fine aggregate performance of the 40%, 50%, and 60% samples. The 28-day high compressive strength with the raw ground-nut shell was 6.1 N/mm^2^ maximum, as needed by the technical standard. Samples made from 40%, 50%, and 60% raw groundnut shells yielded densities of 1.7, 2.2, and 1.9 kg/cm^3^ for groundnut shell (GNS) brick, respectively. A product's mechanical properties meet the IS code standard’s minimum requirements. RSM was then utilized to develop a model for the addition of raw groundnut shell to concrete. R-square and Adeq precision values indicated that the results are highly significant, and equations for predicting compressive strength, water absorption, and dry density have been developed. In addition, optimization was performed on the RSM findings to determine the efficiency optimization of the model. Following the optimization results, experiments were conducted to determine the applicability of the optimized model.

## Introduction

Areas for potential waste made of organic materials produce one of the best pressing issues for developing countries today. Countries in the developing world dispose of their waste by dumping it in landfills, harming the environment. These byproducts also attract many insects, which pose a health risk to humans by spreading a variety of diseases. This study aimed to incorporate organic waste into the construction industry and use it as a low-cost building material^1,2^.

Agricultural waste is frequently used as a component in the production of earth bricks. As a result of incorporating agricultural waste into earth brick, it has greater dimensional stability, strength, and resistance to erosion^3–5^. In addition, the use of agricultural byproducts reduces the thermal conductivity of bricks while also increasing their durability, allowing for the construction of habitats that are more comfortable in cold weather^6–9^.

Previous review studies show that most work has been incorporated into oil palm shells and concrete coconut waste. Therefore, this article reviews the potential to utilize nut waste in producing bricks, mortar, and concrete^6,10,11^.

Studying environmental sustainability in the agricultural industry in India is the primary goal of this investigation. Reusing residues in the construction industry (cement bricks/blocks) is one way to do this environmentally friendly.

The majority of statistical modelling, prediction, and optimization of material properties utilize multivariable regression analysis. In construction and building applications, response surface methodology (RSM) is preferable, especially when multiple variables are involved. This is because RSM can use fewer experiments to evaluate the effects of one or more input variables on a response or properties, thereby reducing time and cost, developing model equations to predict the examined properties using as many variables as possible, and carrying out optimization to determine the optimal dosage of the variables that will yield maximum performance. Adamu et al. utilized RSM to develop a model and optimize pervious concrete mixtures by minimizing durability performance with RHA and CCR as variables. Siamardi et al. adopted RSM for experimental design and model development for the prediction of lightweight self-compacting concrete properties. Their variables included light expanded clay as a coarse aggregate additive and superplasticizer as a cement weighting additive. Kankia et al. developed RSM-based mix design models for fly ash-based geopolymer mortar containing sludge ash from petroleum sludge.

This research can be divided into experimental and statistical sections. The chemical, physical, and mechanical properties of raw groundnut shell and the study's raw groundnut shell production percentage. A study was conducted to determine whether compressive strength, water absorption and dry density could be used as building materials. RSM was used to design the experiment and create models for predicting the properties of brick using cement and raw ground nut shells as the independent variables.

### Production of GNS India

In India, groundnut is the most important oilseed crop, accounting for more than half of the country's total area. Chinese groundnut production is expected to reach 17.57 million tonnes between 2016 and 2022, with India coming in second (6.73 million tonnes), Nigeria fourth (4.45 lakh tonnes), Sudan fifth (2.8 million tonnes), and the United States sixth (2.49 million tonnes). Together, these countries will account for 48.80 million tonnes of global groundnut production^12^. Since agricultural and industrial waste is constantly increasing, they are the root of numerous environmental problems that can be alleviated by repurposing them for brick production.

As per the observations presented in this paper, researchers examined the potential environmental benefits of using groundnut shell in the construction industry by looking at how it could be integrated into a cement-free mortar mix while maintaining its structural integrity.

### Reduction of carbon emissions

This research focuses on reducing carbon emissions from resting groundnut shells and agricultural waste dumping. Regulatory processes for waste management in many developing countries are inefficient, which impacts these countries’ air and environmental quality. For example, during the Groundnut or peanut harvest season, farmers were forced to burn the residues because of a lack of tools for waste management. During the harvesting season, groundnut or peanut shells are burned, resulting in air pollution, and combustion products such as volatile organic carbons (VOCs) are released into the atmosphere (VOCs) and CO2 into the atmosphere (CO). An adverse effect on public health and an increase in the amount of “black cloud” pollution are both caused by this effect^13–17^.

## Related studies

Some reviews have been conducted on using various forms of groundnut shell waste to manufacture concrete.

Previous research^18^ researched using groundnut shell ash (2–10%) as a partial replacement in making light clay bricks (600–850 °C). From a replacement level of 0–8%, the results showed that the average sampling density increased steadily (1500–1225 kg/m^3^), while the water absorption value increased (13–25%); after this, the density increased at 1384 kg/m^3^, and the absorption of water decreased by 18% to 10%. The compression resistance of the groundnut shell ash mixed samples ranged from 7 MPa (8% GSA) to 17 MPa (4% GSA). The most significant amount of SiO_2_ in the groundnut shell ash helped stabilize brick clay, contributing to the increased force. In addition, the flexural resistance significantly decreased with increasing replacement levels. For the control sample, the maximum flexural resistance was measured at 0.13 MPa, while for the groundnut shell ash samples, the optimal resistance was measured at 2% replacement, yielding a value of 0.11 MPa.

A previous work^19^ adopted a mix of 1:5 to replace cement with groundnut shell ash (2–10%). As a result of the ashes, the mortar fixation time was prolonged, indicating its importance in hot weather concreting. The addition of groundnut shell ash resulted in a reduction in density (from 2287 to 2152 kg/m^3^), water absorption (from 9.19 to 7.83%), and compression resistance values (from 3.31 to 2.69 MPa) for 28 days. The incorporation of ashes delayed the time it took for the mortar to set, indicating its importance in hot weather concreting. Adding groundnut shell ash reduced the 28-day density from 2287 to 2152 kg/m^3^, water absorption from 9.19 to 7.83%, and compression strength from 3.31 to 2.69 MPa. It is because of the GSA's lighter weight that the density decreases.

Previous research^20^ experimented with groundnut shell ash to find the best percentage of cement replacement for a given project (15, 20, 30, and 40%). The optimal compression strength of 7 days was reached at 15%, replacing an 8.50% increase from the control sample. After 40% replacement, the force decreased. In regard to groundnut shell ash raw materials, this study looked into using a cement brick. Water absorption, water density and microstructural analysis all confirm these results.

In this research, novelty of excremental work of concrete brick was used as a raw and groundnut shell used as a fine aggregate.

## Materials and methods

### Methodology

This experiment was carried out using raw groundnut shells, cement and water. The cement has a bulk density of 1362 kg/m^3^ and a specific gravity of 3.15, which indicates a high-quality product. In India, agricultural wastes such as groundnut shells were chosen because of their widespread availability. Sieve analysis was conducted to determine whether this local river sand compares with the raw grinded groundnut shells shown in Fig. 7 based on IS 383-1970 standards. Finally, the results were predicted with RSM analysis to determine the correlation and significance of the combination.

### Mix design

Concrete materials were batch by batch. This project's mix ratio was 60:40%t, and 50:50% and 40–60% were the ratios of cement: raw-grafted groundnut shell in the concrete. This was randomly selected to examine new trends with GNS replacement in sandcrete bricks production. For each combination, 3 samples are cast for each day of compressive strength. In total, 85 brick was cast for compressive strength, density and water abortion.

### Material process

#### Mechanization details

**Figure 1:**
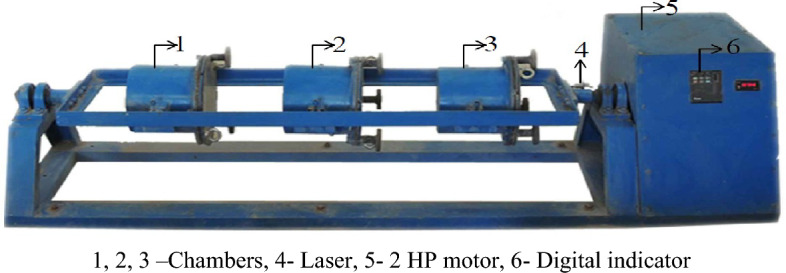
Pulverizer equipment.

**Figure 2:**
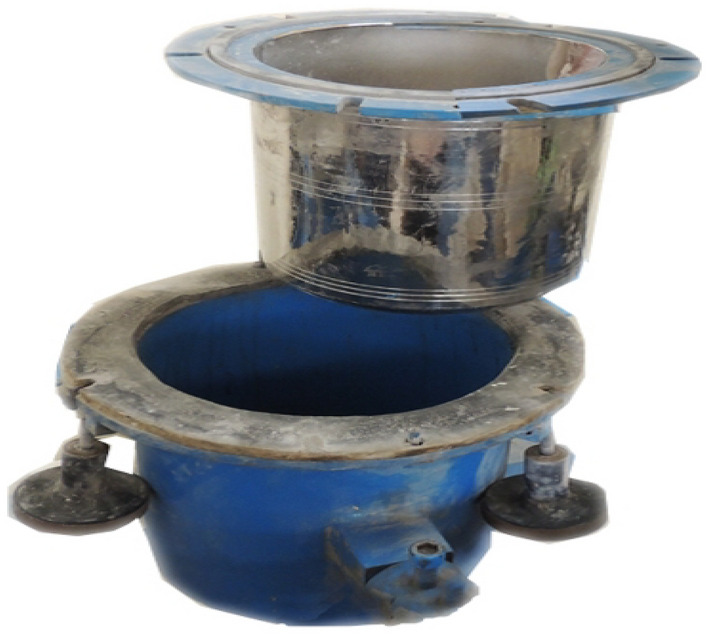
Outer and inner cylinder.

**Figure 3:**
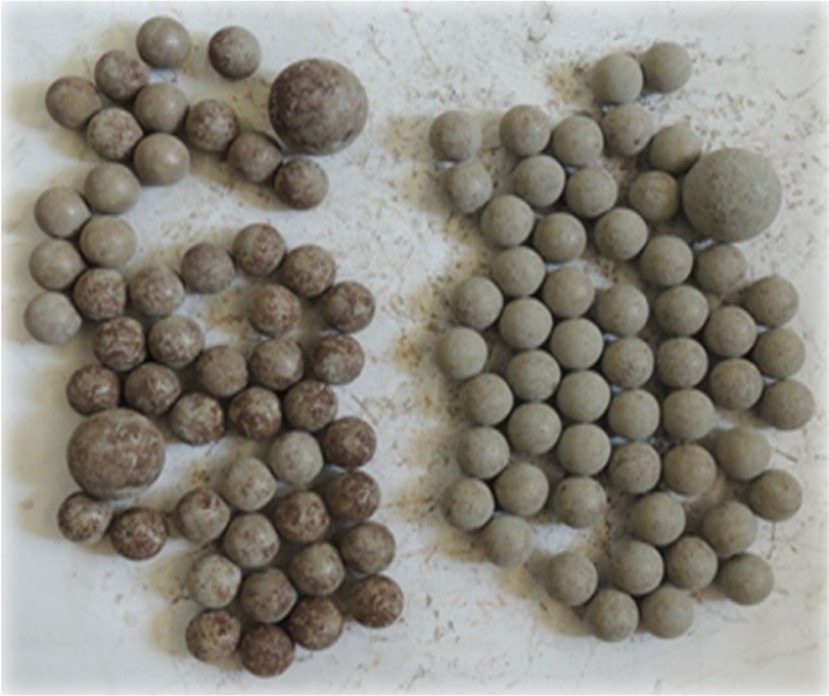
Grinding balls with Φ 12 mm and Φ 25 mm.

After grinding, the raw grinded groundnut shell particles are reduced in size to aggregate particles. Without and with grinding aids, the raw ground nut shell sample was ball-milled. In this process, the fine particles were obtained by grinding with an amine and chloride-based grinding aid. It is possible to produce ultrafine particles with the pulverizer because it has three different chamber numbers, as shown in Fig. 1. A horizontal attrition laboratory pulverizer with a 150 mm diameter and 200 mm length cylinder and 440 °C stainless steel with a dual cylinder model was used to mill the particles, as shown in Fig. 2. Grinders use 20 kg stainless steel balls with 12 mm and 25 mm diameters for the grinding media, as depicted in Fig. 3.

These studies were carried out on samples collected at different grinding time intervals. The low wear rate of the stainless-steel cylinders and balls made them an excellent grinding media choice. Compared to alumina and glass-based grinding media, the efficiency and longevity of carbon-based grinding media are superior.

**Figure 4:**
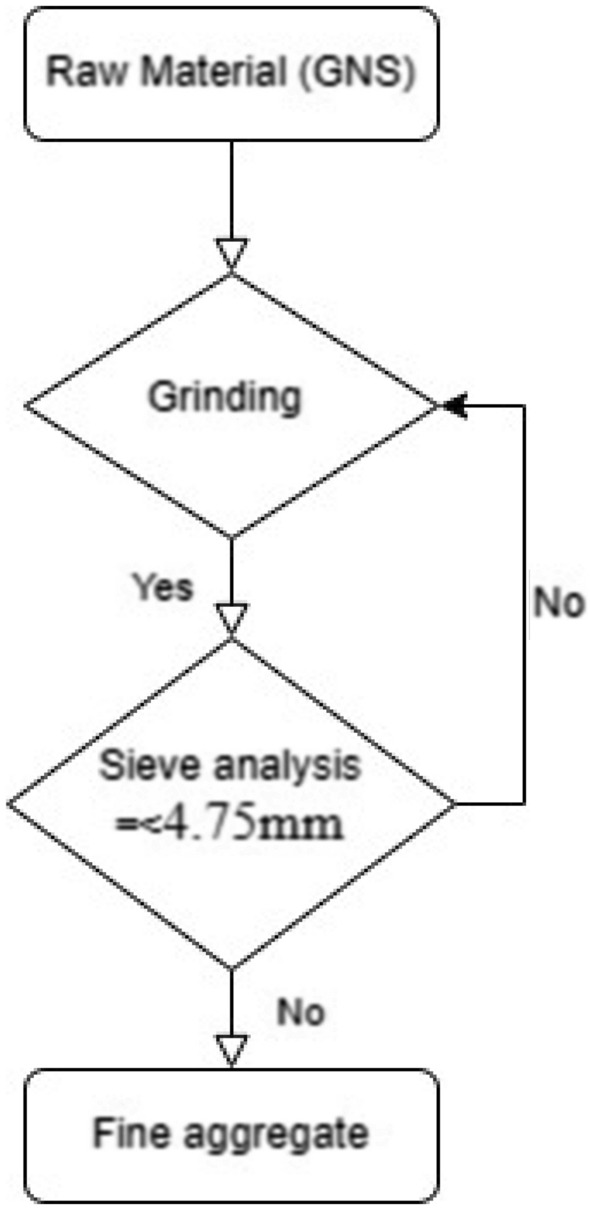
Methodology of grinding process.

**Figure 5:**
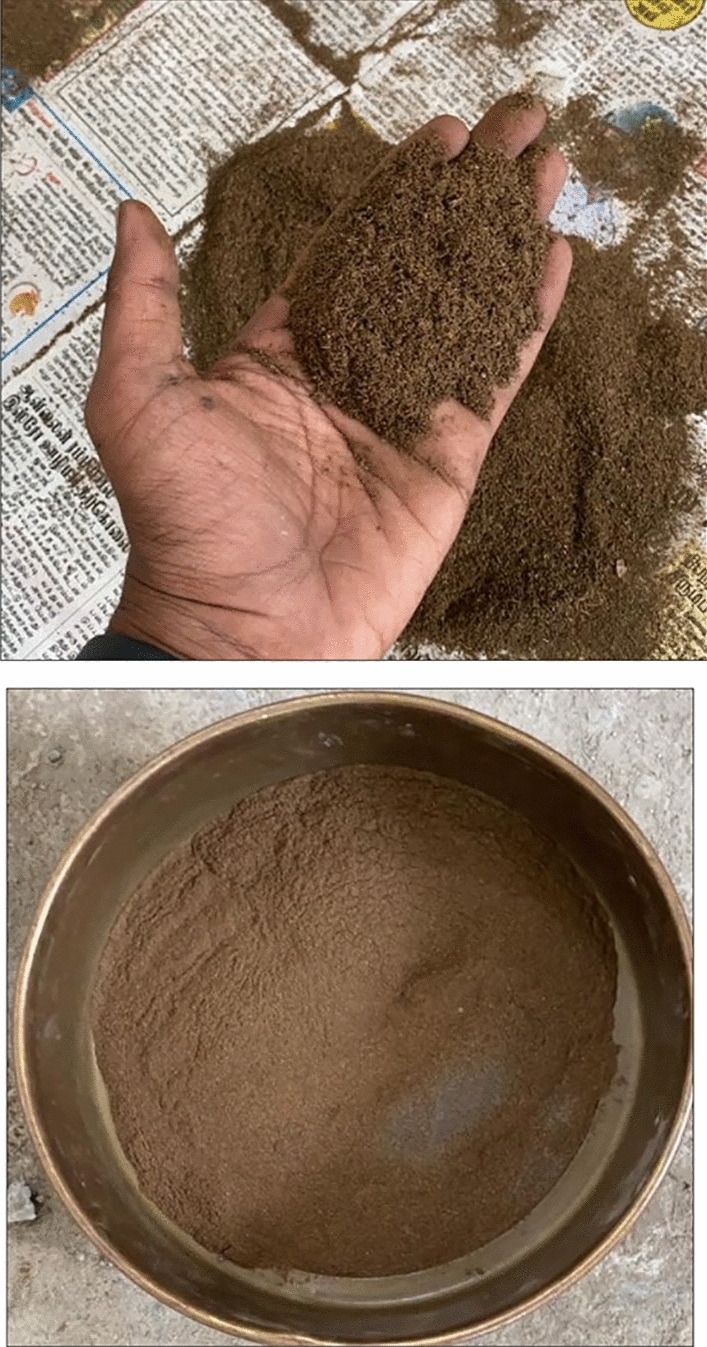
GNS fine aggregate.

**Figure 6:**
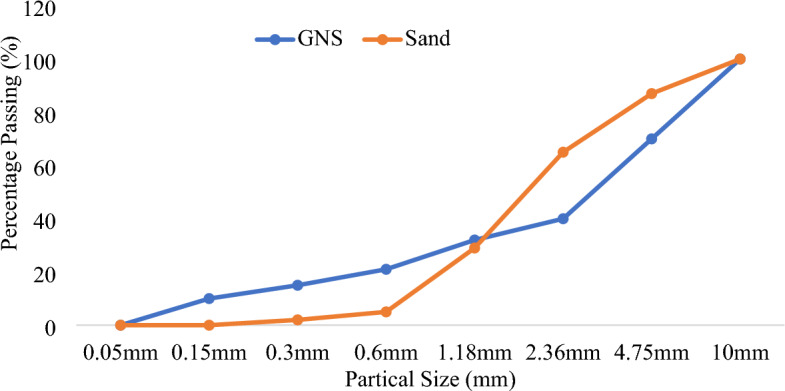
Fine aggregate particle sieve analysis.

The process of transforming GNS into fine aggregates is shown in Figs. 4 and 5. The particle format and size distribution of GNS are both heterogeneous, as shown in Fig. 6. There are three major polymers: hemicellulose, cellulose, and lignin-containing fibres. The chemical and physical GNS components are shown in Tables 1 and 2.

**Table 1:** Chemical properties.

Chemical composition	SiO_2_	Al_2_O_3_	Fe_2_O_3_	CaO	MgO	SO_3_	Na_2_O	K_2_O	P_2_O_5_	MnO_2_	TiO_2_
OPC (%)	17.6	4.02	4.47	67.43	1.33	4.18	0.03	0.39	–	–	–
GNS (%)	26.96	5.82	0.5	9.5	5.6	1.86	1.15	20.02	2	0.32	0.69

**Table 2:** Physical properties.

Physical properties	Cement	Grinded GNS
Bulk density (kg/m^3^)	1362	150
Specific gravity	3.17	1.2
Fineness	0.54	3.49
Water absorption capacity (%)	–	165

#### RSM modelling

RSM is a set of mathematical and statistical techniques used to develop, enhance, and optimize. Typically, the central composite design in RSM is a fractional factorial design employed to determine the functional relationship between the response and independent variables^21^. By computing R^2^, R^2^ adjusted, and R^2^ predicted, we were able to assess the statistical significance of the model developed using the RSM technique. The F value provides insight into the significance of the factors on the observed outcomes. Parameters' effects on the experiment are more noticeable when their associated F values are greater. If the result of the P value is less than 0.05, the significance of the model or parameters is accepted^22,23^.

After conducting experiments, data from the design were analysed to create multivariate regression equations as mathematical models for foretelling concrete qualities. An RSM polynomial model is given by Eq. (1) where x is the input variable, y is the output variable, β is a vector of unknown constant coefficients referred to as parameters, ε and is a random experimental error assumed to have a zero mean^24^. The preference for a quadratic model within response surface methodology (RSM) is driven by its ability to capture nonlinearity, curvature, and intricate variable interactions, enhancing accuracy in system representation. This selection is critical for precise optimization and prediction^25^.

1 $$Y={\beta }_{0 }+{\beta }_{1}A+{\beta }_{2}{B+\beta }_{12}{AB+\beta }_{11}{{A}^{2}+\beta }_{22}{B}^{2}  $$

where *Y* is the predicted response function; *β*_0_ is the intercept; *β*_1_ and *β*_2_ are linear effect coefficients; *β*_11_ and *β*_22_ are quadratic effect coefficients; and *β*_12_ is the interaction effect coefficient.

## Theoretical framework

### Compressive strength

The compressive strength of a lightweight concrete brick is determined by assessing the maximum load in N and the gross cross-sectional area in mm^2^. For these bricks, the gross area is defined as the total size of the brick when it is oriented perpendicular to the load direction. This definition applies to areas within voids and between re-entrant spaces as well.

### Dry density

This investigation needed the preparation of 150 control samples. Each type of waste was made into 50 bricks for each mortar mix proportion (60:40, 50:50, 40:60—cement:GNS waste). The compressive strength, density, and water absorption rate were all measured using three cement blocks for each mixing ratio, with three bricks for each ratio. To conduct the testing, cement blocks were placed under an open shed for up to 28 days to cure. Moisture levels were 90 percent and 27 °C during the curing period in the laboratory.

### Water absorption

The water absorption rate of biobricks was assessed following IS guidelines. After drying biobricks at 105 °C (noted as W1), they were soaked in room-temperature distilled water for 24 h. Following removal and surface drying, the weight (W2) was measured within 5 min. The water absorption rate can be calculated using the specified equation.

$$ {\text{Absorption}}\;(\% ) \, = 100\;({\text{W}}_{2} {-}{\text{ W}}_{1} ){\text{/W}}_{1} . $$

## Results and discussion

**Figure 7:**
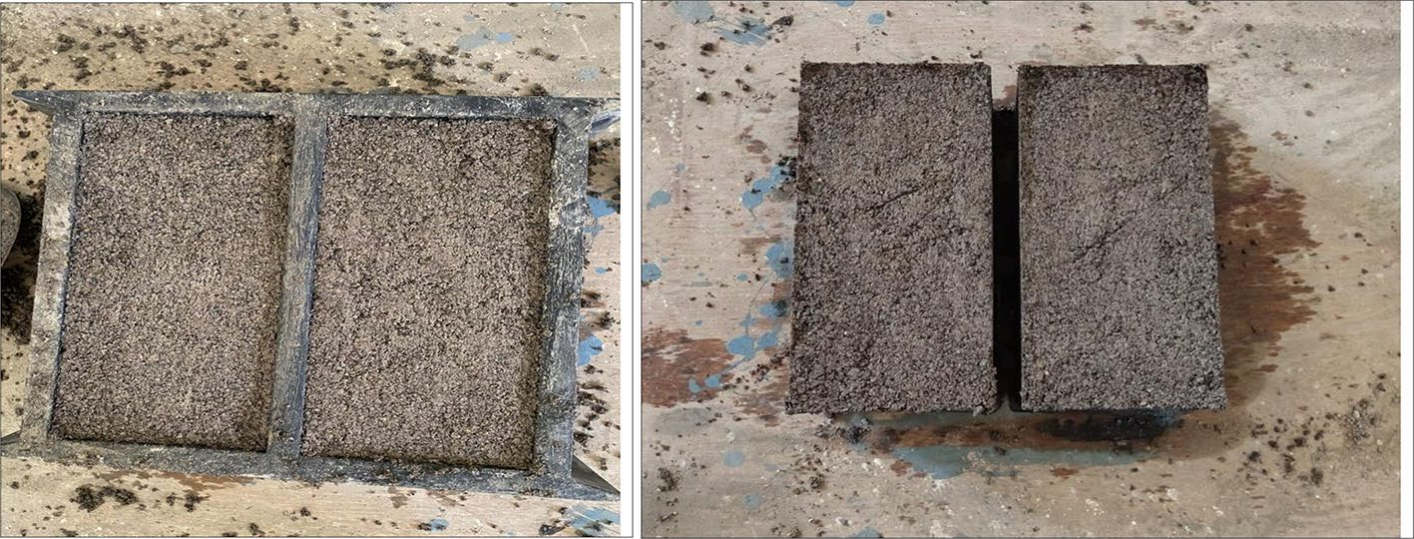
Brick manufacturing.

An effort is made in this section to incorporate raw groundnut shells into the production of building blocks. Cement brick, a common building material in India, is the material of choice. Afterwards, the raw ground nut shell is processed, as shown in Fig. 7. Cement fine aggregate ratios of 60:40, 50:50, and 40:60 is common in cement brick manufacturing, while the water-cement ratio is 0.5. A rotatory mix is used to combine all of the ingredients, followed by the moulding process, which takes place in plastic moulds with an interior dimension of 230 × 110 × 100 mm, as depicted in Fig. 7.

### Density

**Figure 8:**
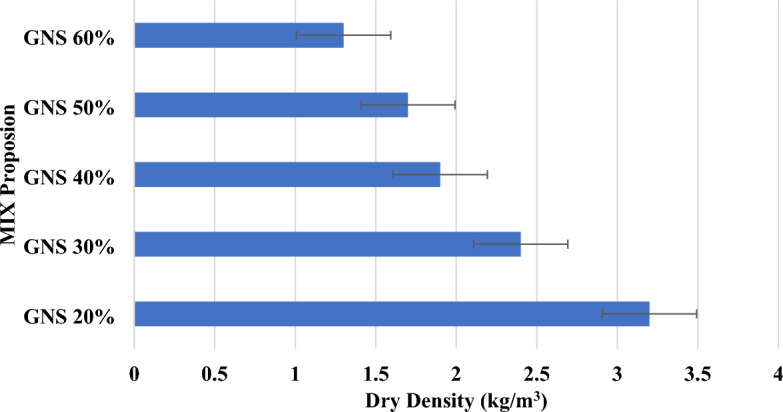
Density of raw groundnut shell brick.

Materials used, cement-to-water ratio, types of sand used and their content, waste material replaced, amount of water absorbed, and so on all affect density^26^. The results show that agricultural waste materials have a lower unit weight than other materials, so their density decreases. Aggregate waste has a low specific gravity, contributing to its low density. With each proportion of waste material, as shown in Fig. 8, according to the dry density of the cement block, the dry densities of the control blocks are very close to the normal weight ranges. There are three types of lightweight blocks: those containing 60%, 50%, and 40% of each waste material. These results corroborate with the results of previous works, which had used agro-waste ashes^5^, seagrass fiber^6^ and cotton and textile ashes^10^ in sandcrete bricks.

### Compression test

Cement blocks were tested for 28 days for compressive strength using the Indian standard (IS3495(1992))^27^. In addition, the axial loading of 230 × 110 × 100 mm cement blocks was studied^28^. A universal testing machine was used to conduct the experimentation at a 2 mm/min rate under displacement control. Then, a load was applied until the cement blocks cracked, and the toughest load was recorded.

An average of three specimens set the minimum criterion of 3.5 MPa as per the Indian standard^29^. The matrix strength, aggregate particle strength, cement content, and water/cement ratio all affect the compressive strength. According to previously published research from a variety of scientists, this is the case. In every experiment, the compressive strength decreased as the percentage of aggregate replacement by agricultural waste materials increased. Using 15% peanut shell as coarse aggregates reduced the concrete's compressive strength by 24%. When groundnut shells were used in place of fine aggregates in a 1:2:3 mixture, the compressive strength was reduced by 49% and 64% compared to control concrete when groundnut shells replaced 25% and 50% of the sand, respectively.

**Figure 9:**
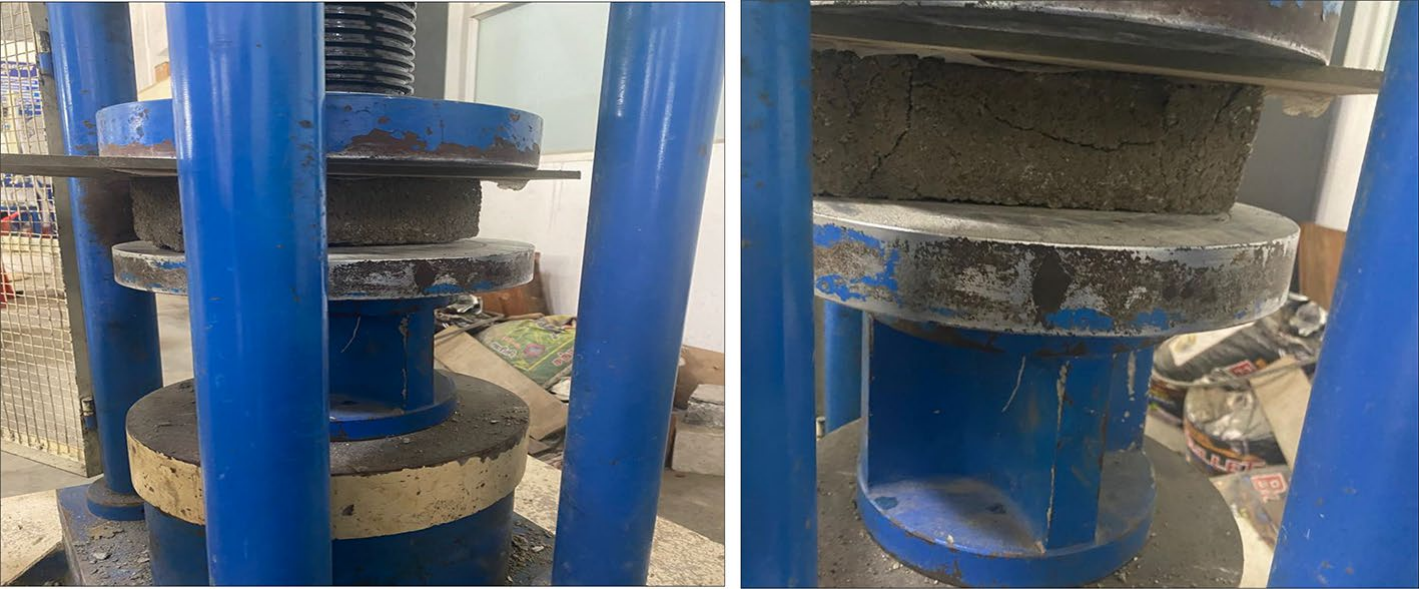
Compressive strength.

**Figure 10:**
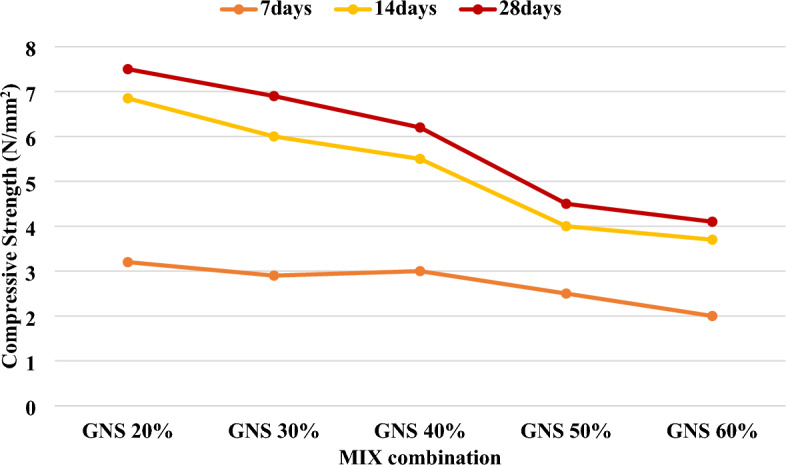
Compressive strength of raw grinded groundnut shell brick.

Here, the compressive strength of the cement blocks used in this experiment can be seen. The compression strength of the partnership decreased as the number of waste containers increased over time in the general trend^30,31^. The higher water/cement ratio used in agricultural added mortar compared to the reference mortar and an increase in agricultural waste content with a much lower mechanical resistance as a sand replacement could explain the results^32,33^. As shown in Fig. 9, the low density of agricultural waste compared to river sand may also reduce its compressive strength. GNS-treated cement blocks have a higher compressive strength than cement and waste material, as demonstrated in this experiment. As a result, according to the India Standard, each type of cement block, except 60:40 raw groundnut shell, meets the specified minimum requirement shown in Fig. 10. These results corroborate with the results of previous works, which had used palm waste^11^, agro-waste ashes^5^, seagrass fiber^6^ and cotton and textile ashes^10^ in sandcrete bricks.

### Water absorption rate

**Figure 11:**
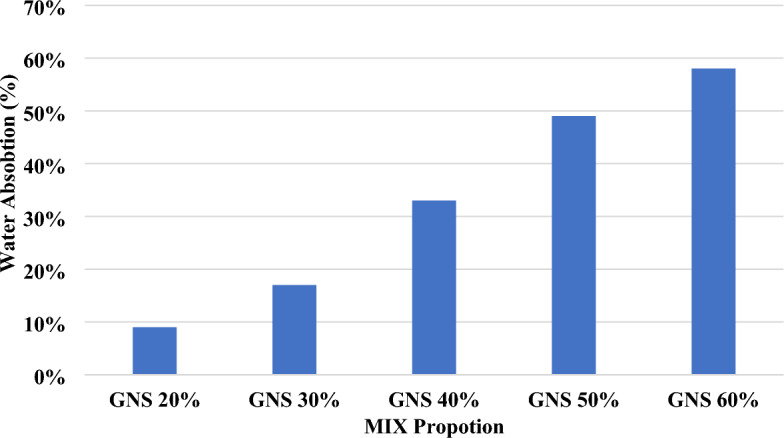
Water absorption of raw grinded groundnut shell brick.

The pore system structure and composition influence the water absorption rate of cement blocks. The waste material's high-water absorption is the primary drawback to its use as a sand substitute in cement blocks. Cement blocks that have been mixed with waste material tend to absorb more water than those that have not been incorporated^34^. Increased water absorption can be seen in previous studies by researchers who replaced fine or coarse aggregates with agricultural waste^35^. Compared to control concrete, the absorption rate of returned waste oyster shell sand increases by 1.1–1.6%^36^. A minimum of three control specimens are cast with the same proportions and cured for 28 days in water to compare results. Figure 11 shows that the water absorption rate of the blocks increases as the amount of waste material increases. Raw ground-nut shell blocks mixed 60:40 control water absorption at a rate greater than the allowed value, according to the results.

### Microstructural properties

**Figure 12:**
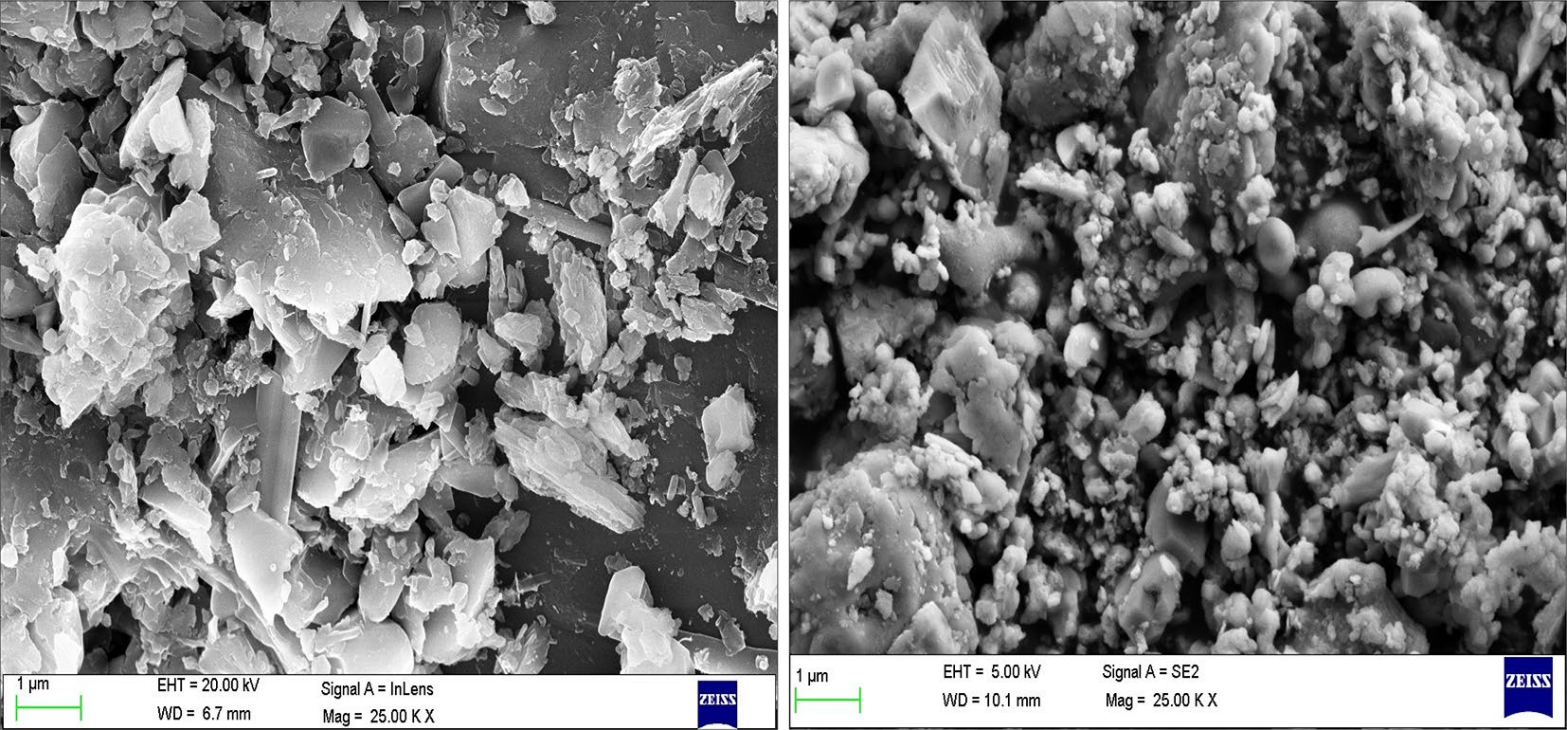
SEM for raw grinded groundnut shell concrete brick.

In Fig. 12, SEM images of a raw grinded groundnut shell concrete brick are shown at different magnifications. The size of the raw grinded groundnut shell concrete brick is smaller than 20 mm, and the raw groundnut shell that expanded in an elongated accordion-like form is shown in the SEM image.

### Response surface methodology (RSM)

**Figure 13:**
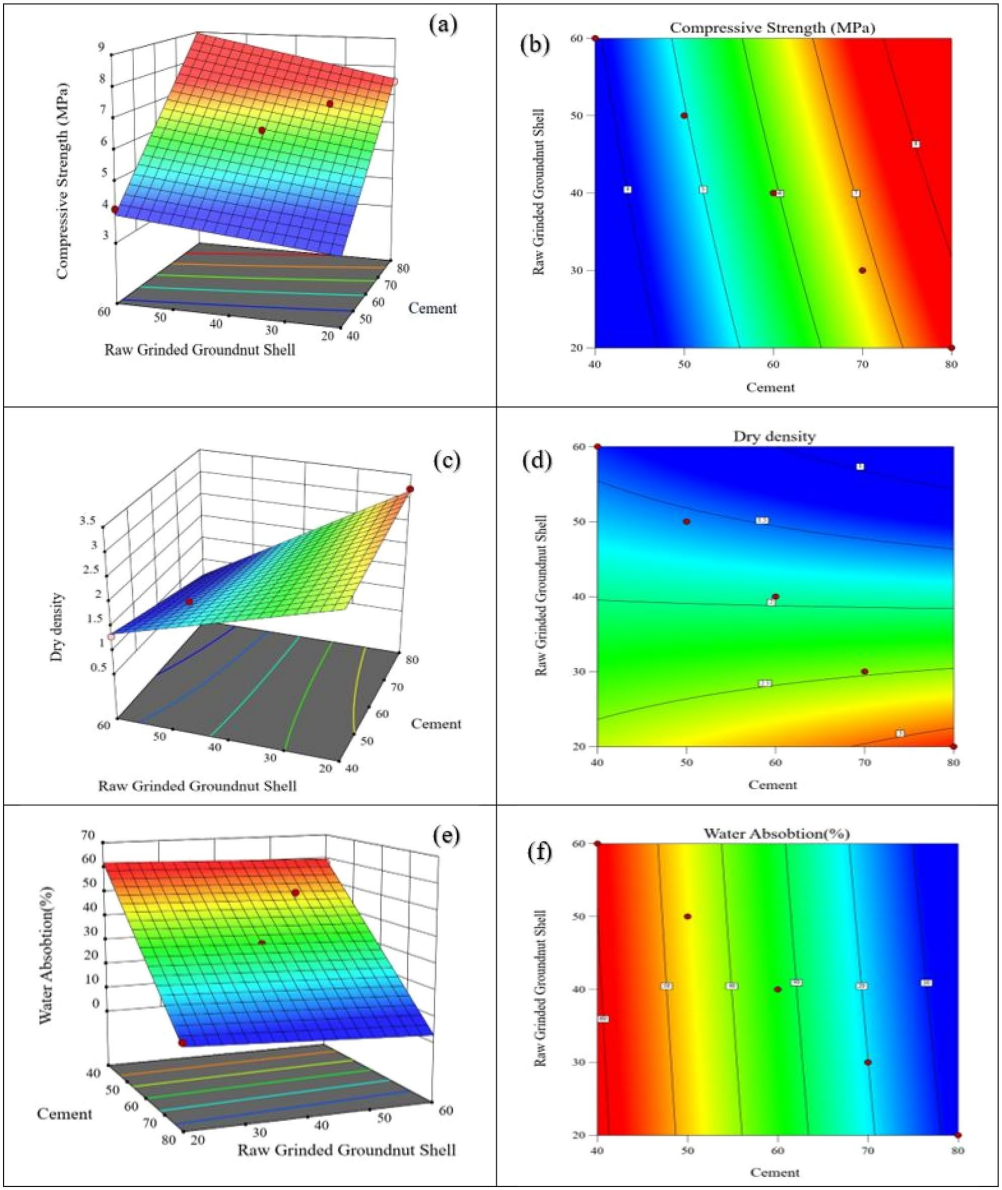
RSM surface plots and counter plots of (**a**) compressive strength surface plots, (**b**) compressive strength counter plots, (**c**) water absorption surface plots, (**d**) water absorption counter plots, (**e**) density surface plots, and (**f**) density counter plots.

**Figure 14:**
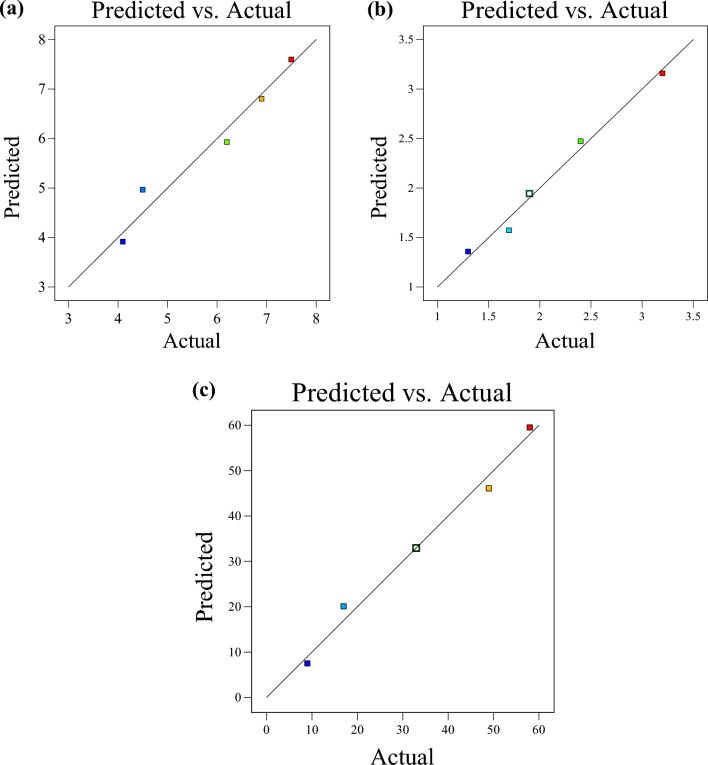
Predicted VS actual (**a**) compressive strength, (**b**) dry density, (**c**) water absorption.

Through the use of overlapping contours, the desired values or a tolerable error band could be attained. Table 3 shows an input of RSM in Design Export. As shown in Fig. 14, multiple responses were taken into account at once, allowing for a realistic range of possible responses. With all other variables held constant, Fig. 13 displays the feasible region with surface plots and contour plots for the responses based on cement and raw groundnut shells.

**Table 3:** RSM design.

Study type	Response surface	Subtype	Randomized
Design model	Quadratic	Runs	5

The significance of the compressive strength model is indicated by its F value of 24.80. There is only a 3.88% possibility that an F value of this magnitude could be caused by noise. The significance of the dry density model is indicated by its F value of 73.90. There is only a 1.34% chance that such a high F value could be caused by noise. The significance of the water absorption model is indicated by its F value of 75.08. There is only a 1.31% chance that such a high F value could be caused by noise.

Table 4 shows the coefficient correlation of the independent variables (cement and raw groundnut shells) and dependent variables (compressive strength, dry density and water absorption). The results confirm that the compressive strength R^2^-0.9612, water absorption R^2^-0.9869, and dry density R^2^-0.9866 are significant for this correlation of independent and dependent inputs and outputs. Regression Table 3 shows the results of Std. Dev., Mean, C.V.%, R^2^, Adjysted R^2^, Predicted R^2^, Added Precision for Compressive Strength, Water Absorption and Dry Density. In Eqs. (2), (3), and (4), equations for predicting compressive strength, water absorption, and dry density are developed.

2 $$ {\text{Compressive}}\;{\text{strength}}\;({\text{N/mm}}^{2} ) \, = \, - 1.13714 \, + 0.100571{\text{C }} + 0.000429{\text{C}}*{\text{ RGGS}} $$

3 $$ {\text{Water}}\;{\text{absorption}}\;(\% ) = \, + 116.05714 - 1.32857{\text{C}} - 0.001429{\text{C }}*{\text{ RGGS}} $$

4 $$ {\text{Dry}}\;{\text{density}}\;({\text{kg/m}}^{3} ) \, = \, + 2.07143 + 0.029286{\text{C }} - 0.000786{\text{C }}*{\text{ RGGS}} $$

where C—cement, RGGS—raw groundnut shell.

**Table 4:** Regression—ANOVA.

	Compressive strength (N/mm^2^)	Water absorption (%)	Dry density (kg/m^3^)
Std. Dev	0.4137	3.36	0.1195
Mean	5.84	33.20	2.10
C.V. %	7.08	10.11	5.69
R^2^	0.9612	0.9869	0.9866
Adjusted R^2^	0.9225	0.9737	0.9733
Predicted R^2^	0.5276	0.7722	0.7886
Adeq precision	11.4840	20.0085	19.4422

#### Diagnostic statistical

This diagnostic statistical graph compares the experimental results on the horizontal axis to the regression model predicted values on the vertical axis to evaluate the generated model's performance in terms of prediction accuracy. The regression line is used to assess well the datasets match the square root transformation output (λ = 0.5) of the response variables, as depicted in Fig. 14. The essence of this analytical diagnostic calculation is to investigate the experimental design in which the developed regression model accurately predicts and evaluates the actual and predicted response relationships. The obtained plot results indicated predicted and actual points ranging from 3 to 8 for compressive strength, 1 to 3.5 for dry density, and 0 to 60 for water absorption.

**Figure 15:**
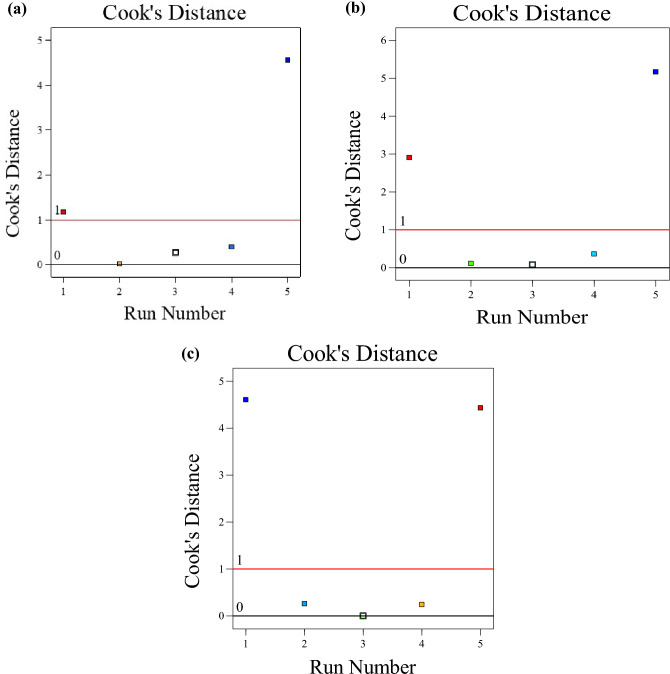
Cook's distance (**a**) compressive strength, (**b**) dry density, (**c**) water absorption.

Cook’s distance is a statistical diagnostic influence tool utilized in regression statistics to determine significant outliers in the factor levels with respect to the response parameters. In addition, it is used to identify regions with a strong apparent correlation, as shown in Fig. 15. The y-axis of the graph of Cook's distance value ranges from 0 to 5, while the x-axis displays. For the compressive strength, dry density, and water absorption responses, experimental run number 1 was observed to lie above the zero to one Cook's distance boundary, indicating that the factor levels have positive effects on the developed model as a whole.

**Figure 16:**
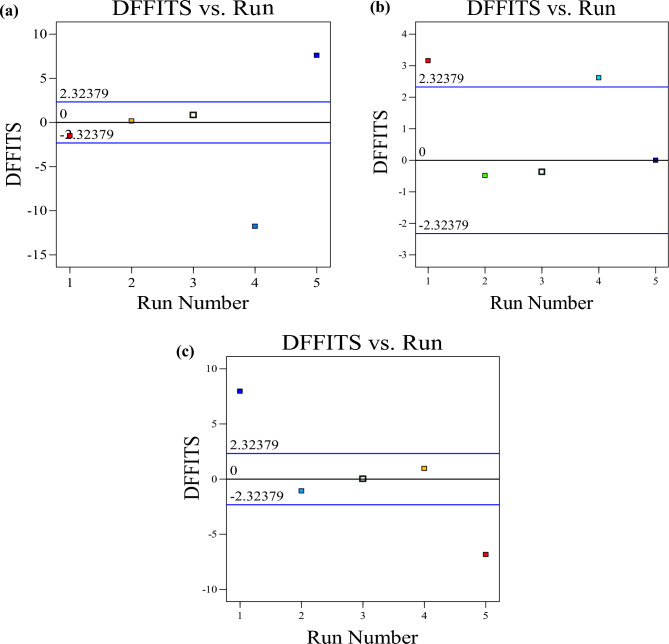
Experimental run vs DFFITS (**a**) compressive strength, (**b**) dry density, (**c**) water absorption.

The DFFITS statistic, which provides a scaled assessment of the expected response variation for the ith observation, is a crucial tool for studentized diagnostic impact. The difference in fits (DFFITS) also defines the variances in the model responses for a particular point on the experimental design when the model fitting processes are excluded, as shown in Fig. 16. The results for compressive strength, dry density, and water absorption were plotted at approximately − 15 to 10 points, − 3 to 4 points, and − 10 to 10 DFFITS points, respectively.

Tables 5, 6, and 7 display the diagnostic statistical summary and influences for the analytical computations performed in this experimental investigation and regression model development for the compressive strength, dry density, and water absorption responses. The results display the predicted vs actual values, the Cook's distance, and the influence on the fitted value.

**Table 5:** Diagnostic statistics for compressive strength.

Run order	Actual value	Predicted value	Cook’s distance	Influence on fitted value DFFITS
1	7.50	7.59	1.174^a^	− 1.510
2	6.90	6.80	0.017	0.165
3	6.20	5.93	0.269	0.840
4	4.50	4.96	0.392	− 11.767^a^
5	4.10	3.91	4.555^a^	7.599^a^

^a^Exceeds limits.

**Table 6:** Diagnostic statistics for dry density.

Run order	Actual value	Predicted value	Cook's distance	Influence on fitted value DFFITS
1	3.20	3.16	2.90^a^	3.157^a^
2	2.40	2.47	0.112	− 0.484
3	1.90	1.94	0.079	− 0.367
4	1.70	1.57	0.363	2.615^a^
5	1.30	1.36	5.16^a^	0.000

^a^Exceeds limits.

**Table 7:** Diagnostic statistics for water absorption.

Run order	Actual value	Predicted value	Cook's distance	Influence on fitted value DFFITS
1	9.00	7.49	4.604^a^	7.967^a^
2	17.00	20.06	0.260	− 1.072
3	33.00	32.91	0.000	0.024
4	49.00	46.06	0.241	0.965
5	58.00	59.49	4.432^a^	− 6.839^a^

^a^Exceeds limits.

## Discussion

The study investigated the impact of various factors on the density and compressive strength of lightweight concrete bricks. Materials such as the type of waste, cement-to-water ratio, sand characteristics, and water absorption were analysed. It was observed that agricultural waste materials contributed to a lower unit weight and decreased density due to their specific gravity. Proportions of waste materials in cement blocks, as depicted in Fig. 10, closely aligned with normal weight ranges. Three categories of lightweight blocks containing 60%, 50%, and 40% waste material were examined.

Compressive strength, a vital property, was influenced by matrix strength, aggregate particle strength, cement content, and water/cement ratio, consistent with previous research findings. The introduction of agricultural waste materials as aggregate replacements led to a decline in compressive strength. For example, substituting 15% peanut shells reduced strength by 24%, while replacing 25% and 50% sand with groundnut shells resulted in reductions of 49% and 64%, respectively.

The water absorption rate of the cement blocks was influenced by the pore structure and composition. Waste materials, known for their high water absorption, were found to increase the blocks' water absorption compared to control specimens. This phenomenon was consistent with previous studies, especially when fine or coarse aggregates were replaced with agricultural waste. Notably, the water absorption rate increased with higher waste material content, exceeding the allowable limit in the case of raw groundnut shell blocks at a 60:40 ratio. In conclusion, the research highlights the complex interplay of various factors affecting the density and compressive strength of lightweight concrete bricks. This underscores the importance of careful material selection and proportions to meet desired standards and mitigate water absorption issues when incorporating waste materials.

## Conclusion

This study aimed to examine the feasibility of using recycled agricultural waste as an alternative to sand as a building material in rural India. In addition, the use of raw ground nut shells in cement blocks was investigated in this study.

- According to IS Standards, cement blocks with a 40:60 and 50:50 mix proportion are considered light cement blocks based on their density. In terms of weight and density, a 60:40 ratio of cement blocks is considered medium-heavy.
- The water absorption rate of cement blocks containing peanut shells is lower than that of blocks containing other agricultural waste.
- As a result of these findings, raw grinded groundnut shell cement blocks made from agricultural waste meet the IS standard's strength requirements. The low strength of the cement blocks allows them to be used in construction.
- The density of the cement brick was reduced by 15% when raw groundnut shell was added, lowering the overall structure's weight. The use of GNS cement bricks can also significantly impact India's waste and construction industries' future, as it aids in achieving positive outcomes in terms of the environment and social and economic aspects of construction in India.
- To analyse the datasets, statistical analysis, ANOVA, and diagnostic statistics were used. By applying the desirability function, numerical and graphical optimization was performed to identify the optimal points of the mixture components with the highest response to meet or satisfy the many outlined requirements for the variables.
- In addition, using raw groundnut shells in Indian building materials will reduce the cost of building materials, which will lower the cost of apartments and solve one of the most pressing issues faced by Indian youth today.

Future study on mechanical properties of (flexible, elastic modulus) durability and prediction of shrinkage.

## Data Availability

The datasets used and/or analysed during the current study are available from the corresponding author upon reasonable request.
